# Heritable variation in colour patterns mediating individual recognition

**DOI:** 10.1098/rsos.161008

**Published:** 2017-02-22

**Authors:** Michael J. Sheehan, Juanita Choo, Elizabeth A. Tibbetts

**Affiliations:** 1 Department of Neurobiology and Behavior, Cornell University, Ithaca 14853, NY, USA; 2 Department of Ecology and Evolutionary Biology, University of Michigan, Ann Arbor 48190, MI, USA; 3 Okinawa Institute of Science and Technology, Okinawa 904-0495, Japan

**Keywords:** individual recognition, negative frequency-dependent selection, animal model, genetic architecture, paper wasps, colour patterning

## Abstract

Understanding the developmental and evolutionary processes that generate and maintain variation in natural populations remains a major challenge for modern biology. Populations of *Polistes fuscatus* paper wasps have highly variable colour patterns that mediate individual recognition. Previous experimental and comparative studies have provided evidence that colour pattern diversity is the result of selection for individuals to advertise their identity. Distinctive identity-signalling phenotypes facilitate recognition, which reduces aggression between familiar individuals in *P. fuscatus* wasps. Selection for identity signals may increase phenotypic diversity via two distinct modes of selection that have different effects on genetic diversity. Directional selection for increased plasticity would greatly increase phenotypic diversity but decrease genetic diversity at associated loci. Alternatively, heritable identity signals under balancing selection would maintain genetic diversity at associated loci. Here, we assess whether there is heritable variation underlying colour pattern diversity used for facial recognition in a wild population of *P. fuscatus* wasps. We find that colour patterns are heritable and not Mendelian, suggesting that multiple loci are involved. Additionally, patterns of genetic correlations among traits indicated that many of the loci underlying colour pattern variation are unlinked and independently segregating. Our results support a model where the benefits of being recognizable maintain genetic variation at multiple unlinked loci that code for phenotypic diversity used for recognition.

## 1. Introduction

Many species depend on individual recognition for tasks with important fitness consequences such as allocating parental care [1], mediating territorial interactions [2] and navigating social hierarchies [3]. Individual recognition requires that the phenotypes of individuals in the population be distinctive; otherwise discrimination would not be possible. Classical hypotheses to explain variation in individually distinctive traits have assumed that individuality is the result of neutral processes [4–6]. However, there is now evidence from traits as diverse as alarm calls in marmots [7], cuticular hydrocarbons in beetles [8] and crickets [9], urinary pheromones in mice [10–12], facial variation in humans [13] and coloration patterns in paper wasps [14,15] that individually distinctive traits are signals that have evolved to facilitate efficient identification [16]. To maximally facilitate recognition, selection for identity signalling is expected to maintain phenotypic diversity in multiple uncorrelated traits, increasing the combinatorial diversity of the system [17].

Two mechanisms could produce the elevated phenotypic diversity involved in identity signals. First, selection for identity signalling could lead to directional selection for increased environmental sensitivity in traits used for individual recognition, giving rise to greater diversity in phenotypes [18]. Under a scenario of increased plasticity, traits should show low to no heritability and be associated with rearing conditions. Second, selection for identity signalling may lead to balancing or negative frequency-dependent selection maintaining genetic diversity in traits used for individual recognition [16,19]. The maintenance of identity signal variation via frequency-dependent selection predicts that traits will have moderate to high heritability and be unassociated with the rearing environment.

The genetic architecture of variation in suites of colour patterns varies across taxa. For example, polymorphisms in multiple wing pattern elements in *Heliconius* are controlled by a single highly variable supergene [20,21], whereas variation in coat coloration in deer mice is controlled by independently segregating mutations spread across introns of *Agouti* [22]. Whether or not traits are expected to be genetically correlated or genetically independent is a function of the benefit or disadvantage of combinatorial variation. Whereas selection on correlated suites of traits is expected to lead to a genetic architecture where a small number of linked loci control polymorphism [23], selection on genetically based individuality should lead to variation at multiple unlinked loci [24]. For example, consider the well-documented strategy-signalling polymorphism in side-blotched lizards, where males pursuing different mating strategies also show a suite of coloration differences [25]. In that system, each mating strategy and its associated colour pattern form an optimal combination and individuals with mixtures of trait that would not accurately signal their strategy would probably be selected against. Indeed, studies have documented correlated selection favouring the co-expression of a strategy and a particular suite of colour traits [26,27]. Theory predicts that suites of identity-signalling traits should be uncorrelated or weakly correlated, because lower trait correlations generate greater combinatorial variation in overall appearance [16,17]. Combinatorial variation can greatly increase variation in a population as it allows for individuals to be differentiated along multiple phenotypic axes. For example, the various parts of human faces show lower levels of inter-trait correlations compared to traits on the rest of the body [13]. The lack of correlations among many facial traits in humans leads to greater overall diversity in faces, facilitating recognition. Correlation among traits reduces the number of axes of variation, making distinctions between individuals more challenging. Uncorrelated trait diversity that is expected for individual identity signals could arise either because traits are sensitive to different features of the rearing environment or controlled (at least partly) by unlinked, independently segregating loci.

**Figure 1.**
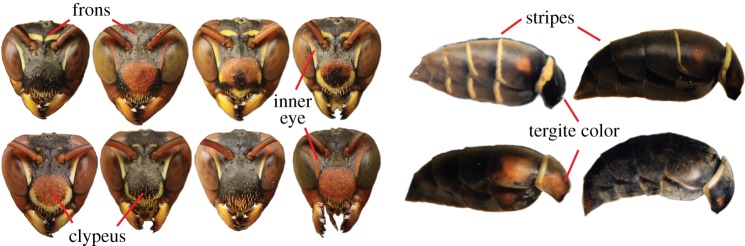
Variable colour traits in *P. fuscatus*. Female *P. fuscatus* have extensive variation in the coloration of their faces and abdomens that is used for individual recognition. We scored variation in five different traits: (i) frons yellow, (ii) clypeus yellow, (ii) number of yellow stripes on the dorsal side of the abdomen, (iv) clypeus black, and (v) the number of dorsal abdominal tergites with brown pigment.

The highly variable colour patterns found in female *Polistes fuscatus* wasps provide an ideal system for examining the mechanisms producing identity signal diversity (figure 1). Colour pattern diversity in *P. fuscatus* has been a source of confusion for taxonomists and others working on *P. fuscatus* for over a century [28–30]. Female wasps of this species show dramatic variability in yellow pterin-based markings and variation in the extent of brown and black caused by melanin in multiple different regions of the body [3]. The colour patterns led to confusion because they are highly diverse; not associated with behavioural strategies or quality; and are largely uncorrelated with each other [3]. More recent research has shown that the colour patterns are used for individual recognition [3,31,32] and that selection for signalling identity is an important force maintaining colour pattern variation within populations of this species [14,15]. Furthermore, behavioural experiments illustrate that there are benefits of rare, identifiable colour patterns [14]. The mechanisms that lead to colour pattern variation have yet to be established.

Here, we investigate the quantitative genetic architecture of the highly variable identity-signalling colour patterns of female *P. fuscatus* paper wasps (figure 1). Specifically, we assess whether there is additive genetic variation underlying trait diversity. High additive genetic variation underlying trait diversity indicates that phenotypic variation arises via the maintenance of genetic variation. Low additive genetic variation underlying trait diversity suggests that variation arises via plasticity with environmental variation explaining a significant portion of individual colour variation. Using a population of wild nests of *P. fuscatus,* we test whether variation in identity-signalling colour patterns is heritable. Given that we find substantial heritability in colour patterning, we also investigate patterns of genetic correlations among traits to examine the extent to which the production of different combinations of traits may be aided or impeded by the genetic architecture of the traits.

## 2. Material and methods

### 2.1. Sampling regime and pedigree construction

We scored colour patterning in foundresses and their offspring on nests in southeastern Michigan from the autumn of 2008 to the autumn of 2011 (*n* = 856 female wasps). Small wooden boxes were placed in fields and along the edge of wooded areas to encourage wasp nesting following previous studies of wasp populations [33,34]. Multiple queens initiated approximately half of the nests that successfully produced offspring, consistent with patterns of cooperative nesting in this species at other locations [35]. In cooperative nests, reproduction tends to be shared among foundresses though skewed in favour of the most dominant individual [36]. We genotyped a small portion of individuals from nests with a range of foundress group sizes to determine whether there was sufficiently high reproductive skew to assign the majority of reproduction to a single foundress (electronic supplementary material). Additionally, this exercise allowed us to examine the extent to which drifting across nests might be a problem for our study. Radio-tracking of workers on the tropical wasp *Polistes canadensis* revealed high rates of workers moving between nests, which would complicate analyses and lead to lower than expected relatedness on nests [37]. Patterns of drifting have not been explicitly studied in *P. fuscatus.* Genotyping confirmed that single-foundress nests represent full-siblingships but that nests with larger number of foundresses had increasingly lower relatedness values among individuals as is expected under the imperfect skew typically seen in *P. fuscatus* populations ([36], electronic supplementary material, table S1). As a result, definitively assigning the mother–offspring relationships on multiple foundress nests would require extensive destructive genotyping of all wasps, which was not possible in our study because it would have decimated our study population. Therefore, we limited our analyses to single-foundress nests (*n* = 83 colonies). Additionally, the fact that single-foundress nests appear to represent full-siblingships means that working drifting, if it occurs, is likely to be a negligible factor in our analyses.

We used the inferred mother–daughter relationships from single-foundress nests to construct a pedigree for our population. Because we marked future foundresses at the end of each summer and in early autumn, we were able to follow the same family lineages for multiple generations. Most of the marked foundresses that returned to our nest-boxes formed cooperative nesting associations, removing them from our analysis. However, for four of the 83 nests included in our analysis we know the maternal nest of origin and these data are included in our pedigree.

### 2.2. Colour pattern analysis

We collected, photographed, marked and then returned wasps to their nests each spring and autumn. From photographs, we scored variation in the presence and extent of five variable colour markings within the population: (i) a yellow stripe on the frons just above the antenna, (ii) a yellow stripe along the edge of the clypeus, (iii) the number of yellow stripes on the dorsal side of the abdomen, (iv) the extent of black on the clypeus, and (v) the number of dorsal abdominal tergites showing brown coloration. With photographs of live wasps, it was not possible to achieve the same orientation and angle in each photograph, making precise quantitative estimates of colour pattern variation challenging. Instead, we scored each colour pattern on an ordinal scale from 0 to 4 (electronic supplementary material, table S2). The scores for the abdominal colour patterns are counts of the number of segments on which the colour occurred.

### 2.3. Estimation of variance components and genetic correlations

To estimate components contributing to phenotypic variation, we used the ‘animal model’ as it is implemented in the R package MCMCglmm [38]. The standard pipeline for analysis in MCMCglmm expects diploid genetics. Because wasps are haplodiploid, we used a different package to estimate the genetic covariance matrix among animals in our pedigree. We estimated the genetic covariance matrix among individuals using the ‘makeS’ function of the nadiv software package in R to estimate sex-chromosome-specific patterns of inheritance [39], which is functionally equivalent to haplodiploidy [40]. Our analysis makes use of the full pedigree of 83 nests. In addition to the relationships between mothers and daughters, the genetic covariance matrix also takes into account the super-relatedness (*r* = 0.75) among full sisters as well as relationships between generations when they are known.

For each trait we considered four models that examined the contributions of different variance components to phenotypic variation: (i) additive only, (ii) additive and maternal, (iii) additive and rearing season, and (iv) additive, maternal and rearing season. For all traits, we classified the trait data as ordinal (scale 0–4). We ran all models with weakly informative priors and our results were robust to changes in these parameters and consistent across multiple runs, as the best model did not change. Models varied considerably in their deviance information criterion (DIC, electronic supplementary material, table S3), and in the main text we report only the result of the model with the lowest DIC, which is the model with the greatest explanatory power [41]. In all but one case, the best performing model had a much lower DIC (ΔDIC > 145) than the next best model. For the extent of black on the clypeus, we report two more similar models (ΔDIC = 15). Values for variance components are reported as the estimates of the mean and the 95% confidence intervals. To determine whether traits were genetically correlated, we ran bivariate animal models using MCMCglmm in R. The code used to run the models in MCMCglmm is provided in the electronic supplementary material.

Under a simple model of Mendelian inheritance, sisterships of wasps would all display a trait, none display a trait or half display a trait. These ratios are derived from the fact that fathers are haploid and thus contribute the same allele to all the daughters and *Polistes* are monogamous [42]. We examined the patterns of inheritance for two colour traits, which could be scored for categorical presence/absence (frons yellow and clypeus yellow) in single-foundress nests with at least 20 offspring. We report the *p*-value for *χ*^2^-tests, assessing whether the patterns observed are significantly different from half of the daughters with and without the trait. In no case was a trait fixed among daughters from a large nest. Additionally, we do not test for significant differences from none or all of the daughters showing a trait, as even one individual with or without a trait would reject a Mendelian prediction of none or all of the offspring showing a trait.

## 3. Results

All five colour traits scored in our analysis have significant components of additive genetic variance (table 1). For the three facial traits, the simplest ‘additive variance only’ model was considered the best model, suggesting that data for these traits are best explained without significant contributions of birth year or maternal effects on phenotypic variation. In all of the facial traits, the best animal models indicate that additive genetic variance accounted for more than 90% of phenotypic variance (table 1). For the amount of black on the clypeus, a model including birth year as a random effect was only slightly worse than the ‘additive only’ model, so we also report that. Even in this case with birth year as an additional variance component, the amount of variation attributable to additive genetic effects is high at 83%, with only 5% of variance attributable to birth year. For both abdominal traits, models with additional variance components were retained as the best-fit model. The number of abdominal tergites with brown markings was best explained by a model with maternal effects. Additive genetic variance explained 69% of phenotypic variance with maternal effects accounting for an additional 17% of variance in the number of segments with brown markings (table 1). The number of abdominal segments with yellow stripes was best explained by a model with birth year as a random effect. Here, additive effects explained 90% of phenotypic variance in the number of yellow stripes, with an additional 3% explained by the year in which a wasp was born.

**Table 1.** Variance components explaining phenotypic variation. (i) Data show the posterior mode (95% confidence interval). (ii) For black clypeus, models are named in order of the lowest DIC value (model 1 < model 2). (iii) Data analysed for 856 female wasps from 83 single-foundress nests.

trait	additive variance	other variance component
yellow frons	0.9993 (0.95–0.99991)	—
yellow clypeus	0.92 (0.88–0.96)	—
black clypeus (model 1)	0.92 (0.88–0.95)	—
black clypeus (model 2)	0.84 (0.66–0.91)	0.05 (0.02–0.26)—birth year effects
abdomen brown	0.69 (0.35–0.94)	0.17 (0.0007–0.41)—maternal effects
abdomen yellow	0.90 (0.71–0.95)	0.3 (6 × 10^−8^–0.22)—birth year effects

Consistent with the variability seen in each trait, the distribution of colour patterns within large single-foundress nests rejects a simple model of Mendelian inheritance when we consider the presence/absence of a trait (table 2). For yellow marks on the frons, we find three instances in which the mother lacked a mark and significantly less than half of the daughters showed the marking, rejecting a Mendelian model. In one case, a single daughter possessed a marking, which raises the possibility that the lone wasp might not actually be a daughter from the nest (i.e. she could have been a drifter from a different nest). While we cannot formally rule out that possibility, we note that the two other instances that reject a Mendelian ratio involve multiple daughters displaying a trait. Collectively, the data from all the nests allow us to confidently reject a Mendelian inheritance hypothesis. For yellow markings on the clypeus, three mothers with markings produced nests in which significantly more than one half (but not all) of the daughters showed the markings, rejecting a Mendelian model.

**Table 2.** Inheritance patterns reject a simple Mendelian single-locus genetic architecture (*P*-values reported here show whether or not nest differences from the 50 : 50 distribution predicted for Mendelian traits in a haplodiploid organism. Significant *p*-values are indicated by italics.)

nest	present in mother	number of daughters with trait	total number of daughters	*p*-value
frons yellow				
2009.bg4	y	10	24	0.53
2009.bg49	*n*	*1*	*22*	*<0.0001*
2009.bg62a	y	11	22	1
2010.e5b	y	8	22	0.29
2010.p1a	*n*	*4*	*32*	<*0.0001*
2011.bg18	*n*	*3*	*34*	<*0.0001*
clypeus yellow				
2009.bg4	*y*	*20*	*24*	*0.0022*
2009.bg49	y	10	22	0.84
2009.bg62a	*y*	*18*	*22*	*0.006*
2010.e5b	y	15	22	0.14
2010.p1a	*n*	22	32	0.052
2011.bg18	*y*	*28*	*34*	*0.0003*

Patterns of genetic correlations among traits suggest that two largely, though not entirely distinct, sets of loci control variation in yellow versus brown markings in *P. fuscatus*. Genetic correlations among traits are reported in table 3. We find moderate genetic correlations among three yellow pterin-based markings (range of genetic correlations: 0.32–0.36). Variation in the melanin-based markings on the clypeus and abdomen (i.e. extent of black versus brown) are also genetically correlated (−0.68). Note that the negative correlation arises because we scored the extent of black on the clypeus and the amount of brown on the abdomen, i.e. more brown means less black and vice versa. Yellow pterin marks on the frons and on the abdomen are not correlated with either melanin-based trait, though the extent of yellow pterin markings on the clypeus is associated with both melanin markings (−0.49 with clypeus black; 0.29 with brown marks on the abdomen).

**Table 3.** Genetic correlations among colour pattern traits. (Significant values where the 95% CI does not include zero are italicized. Data analysed for 856 female wasps from 83 single-foundress nests.)

	yellow clypeus	black clypeus	abdomen brown	abdomen yellow
yellow frons	*0.46* (*0.36–0.59*)	−0.02 (−0.21–0.14)	0.11 (−0.04–0.26)	*0.33* (*0.21–0.50*)
yellow clypeus		*−0.49* (*−0.64–−0.36*)	*0.29* (*0.14–0.42*)	*0.32* (*0.22–0.47*)
black clypeus			*−0.68* (*-0.81–−0.56*)	−0.04 (−0.19–0.17)
abdomen brown				−0.06 (−0.23–0.07)

## 4. Discussion

Our analyses of colour patterning suggest that selection for individual identity signal variation in *P. fuscatus* maintains genetic variation at multiple distinct loci. Animal models using a pedigreed population of wild paper wasps indicate that colour pattern variation has a very strong additive component for most traits. Patterns of continuous phenotypic variation in traits and explicit rejection of a Mendelian model of inheritance suggest that colour pattern traits are polygenic. Finally, we find that some traits composed of different pigments are genetically uncorrelated, indicating that distinct independently segregating sets of loci control coloration patterning. These results combined with previous studies showing evidence of selection for distinctive colour patterning [14,15] support a model in which individual recognition maintains variation at multiple independently segregating loci in paper wasps, while rejecting a model where individuality is the result of increased phenotypic plasticity.

For all but one trait, additive genetic variance accounted for greater than 90% of phenotypic variation. This result correctly captures that colour patterns have a substantial additive genetic basis, but the estimates may be elevated owing to methodological constraints of the study. Notably, the need to return wasps to the wild prevented us from producing standardized photographs to robustly quantify phenotypic differences. The ordinal scoring system in this study bins phenotypic variation and may mask some non-additive modes of inheritance or environmental effects. However, collapsing variation into bins will not in itself create the strong additive genetic component found in our data.

The strong additive genetic component of colour pattern variation is in line with previous work showing that colour pattern development is not sensitive to factors such as larval nutrition during rearing [43]. Two previous studies provided experimental evidence that abiotic conditions can plastically influence melanin-based coloration in paper wasps [44,45], though these effects are seen from large-scale changes in abiotic conditions. Although these previous results may explain variation in coloration across a species' range, they are unlikely to explain variation in coloration within and between neighbouring nests that experience very similar climatic regimes. Rather, our data suggest that there is substantial additive genetic variation underlying colour pattern diversity found within a population. The strong heritable component of wasp colour patterning is consistent with a pattern of highly heritable identity signalling phenotypes found in other taxa such as human faces [46,47] and mouse urinary scents [11,48].

Individuality is the result of combinatorial variation in multiple phenotypes in *P. fuscatus* wasps [3] and other taxa [13,49–52], which has led to the prediction that traits involved in individual identity should be genetically uncorrelated [16,17]. Here we find a mix of traits with mostly moderate genetic correlations (e.g. 0.3–0.5) and traits that are genetically uncorrelated. All the examples of uncorrelated trait pairs are composed of two distinct pigments. In the case of genetically correlated traits composed of the same pigment, it is not surprising that loci might have pleiotropic effects on multiple colour patterns through changes to a shared colour synthesis pathway. For example, changes to a single amino acid in the *MC1R* gene involved in melanin synthesis were found to alter multiple aspects of colour patterning in populations of beach mice [53].

Although we detect moderate genetic correlations among some trait pairs, a substantial portion of the additive genetic variance appears to be specific to a given trait, as most of the variance is not shared between traits. Indeed, distinct mutations are known to influence the deposition of the same pigment on different parts of the body in other systems [22]. The present dataset does not identify the number of loci that may be involved in colour pattern variation in paper wasps, though it does indicate that the genetic architecture of wasp coloration patterning is dependent on variation at multiple independently segregating loci (tables 2 and 3). As a result, wasps may still show substantial combinatorial diversity in trait values despite modest genetic correlations.

In recent years, it has become increasingly clear that colour pattern variation is commonly used for signalling in paper wasps [54]. Unlike *P. fuscatus,* most species of wasps examined so far have been found to use quality signals rather than signals of individual identity [55–61]. Although the evolutionary pressures leading wasps to use quality versus identity information are unclear [62], the developmental basis of trait variation is expected to be very different. Indeed, in *Polistes dominula* where it has been examined most thoroughly, there is experimental evidence that quality signalling colour pattern variation is strongly condition–dependent, with modest heritability detected under some environmental conditions [43,63]. The development of quality signals in other species has yet to be investigated, but will probably show similar patterns. Individual recognition has also been described in *Liostenogaster flavolineata* hover wasps, where colour patterns appear to mediate acceptance and rejection of individuals as nest-mates [55,64]. A recent analysis of colour pattern diversity in *L. flavolineata* found that colour patterns cluster by colony [65], which is consistent with an additive genetic basis of colour pattern variation in that species, though specific estimates of the quantitative genetic architecture are needed. Other species of wasps show complex colour patterns that could mediate individual recognition [66] and might be expected to show similar patterns of genetic architecture as we have found here, though tests for individual recognition and colour pattern heritability are needed in other wasps to understand how generalizable the patterns found in *P. fuscatus* are to colour pattern variability in other social wasps.

These findings build on a growing body of evidence that social interactions can maintain phenotypic variation in traits facilitating recognition [7,9,11,13,67–69]. Our evidence is consistent with the hypothesis that variation is maintained owing to negative frequency on multiple, as of yet unidentified, regions of the *P. fuscatus* genome instead of selection for increased phenotypic plasticity. Thus, social recognition in animals may have unexpectedly broad influences on patterns of genetic diversity within species.

## Supplementary Material

Supplemental methods, tables and R code

## Data accessibility

Data available from the Dryad Digital Repository: http://dx.doi.org/10.5061/dryad.kb60v [70].
