# Supplementary material for: Heritable variation in colour patterns mediating individual recognition
Source: R Soc Open Sci. 2017 Feb 22;4(2):161008. doi: 10.1098/rsos.161008 (PMC5367277; doi:10.1098/rsos.161008)
Supplement: Supplemental methods, tables and R code [file rsos161008supp1.docx]

Supplemental methods, tables and R code for “Heritable variation in color patterns mediating individual recognition”

**Relatedness among wasps on multiple foundress nests**

The level of relatedness among the offspring of multiple foundress nests depends on the relatedness among foundresses and the relative skew in reproduction among the foundresses. At one extreme, a nest with multiple unrelated foundresses, each with an equal share of reproduction will show very low average levels of relatedness. At the other extreme, the daughters of a nest with complete reproductive skew would all be full siblings. Published data suggest that most nests are expected to fall somewhere in between. Most cofoundresses are former nestmates and thus related (Klahn, 1979). At our study site, mark-recapture studies found that in all 26 multiple foundress nests initiated by marked foundresses over a two-year period (2010-2011), the foundresses had been born on the same nest in the previous year. This suggests that multiple foundress nests represent extended families.

We further investigated the relatedness level of multiple foundress nests by destructively genotyping a small portion of offspring from a few nests. We used 12 previously developed primers (Pbe128, Pbe430, Pmet44592, Pbe475, Pmet45548, Pmet40472, Pmet41635, Pbe203, Pbe205, Pmet45195, Pbe269, Pmet46789) to genotype wasps using microsatellites and analyzed pairwise relatedness among individuals using the R package ‘demerelate’ (Kraemer and Gerlach, 2013). This analysis demonstrates that multiple foundress nests are mixtures of families, with foundress groups tending to produce slightly less related families as the number of foundresses increases (Table S1). Since multiple foundress nests represent mixed families, we excluded all multiple foundress nests from our analyses of heritability and genetic correlations since the pedigree was uncertain.

Table S1: Relatedness among offspring in relation to nest size

| Nest | Mean pairwise relatedness | Offspring genotyped | Number of Foundresses |
| --- | --- | --- | --- |
| bg19.2010 | 0.7987 | 5 | 1 |
| r12a.2009 | 0.7553 | 5 | 1 |
| r14b.2011 | 0.6295 | 7 | 1 |
| p9b.2011 | 0.6491 | 5 | 2 |
| bg16.2009 | 0.6424 | 7 | 2 |
| F1.2011 | 0.1647 | 8 | 2 |
| bg6.2009 | 0.6462 | 8 | 3 |
| r9.2011 | 0.5292 | 5 | 3 |
| p7b.2011 | 0.469 | 5 | 3 |
| bg29.2010 | 0.074 | 14 | 3 |
| bg1.2011 | 0.3427 | 23 | 4 |
| P1a.2011 | 0.3035 | 5 | 4 |

Table S2: Ordinal Trait Scoring Rubric

|  | **Trait** | | |
| --- | --- | --- | --- |
| **Score** | **Yellow Frons** | **Yellow Clypeus** | **Black Clypeus** |
| **0** | Absent | Absent | No black observed in the center of the clypeus, a very thin edge of black may be observed |
| **1** | Small, unconnected patches of yellow | Small dot of yellow a the tip of clypeus | Small to medium sized dot of black in the center of the clypeus ( < 0.5 mm2) |
| **2** | Two yellow patchs are connected, but are thin (< 0.2 mm) | Thin line of yellow at the bottom of the clypeus, does not extend all the way to the eye | A band of black stretching across the clypeus, occupying less than 75% of the total area |
| **3** | Thick yellow marks connected at the midline (> 0.2 mm) | Yellow band along the two sides and bottom of the clypeus, resembling a smile | More than 75% of the clypeus area not covered by yellow is covered by black |
| **4** | Very thick yellow marks, connected at the midline (> 0.4 mm) | As with 3, but very thick (>0.4mm) | No brown is present, all area of the clypeus that are not yellow are black |

Table S3: DIC values for all models considered

|  | **Models** |  |  |  |
| --- | --- | --- | --- | --- |
| **Trait** | **Additive variance only** | **Additive + Season** | **Additive + Maternal** | **Additive + Maternal + Season** |
| Yellow frons | **827** | 1115 | 1217 | 1222 |
| Yellow clypeus | **1432** | 1968 | 1680 | 2084 |
| Black clypeus | **1960** | 1975 | 2115 | 2152 |
| Abdomen brown | 1049 | 1051 | **904** | 1109 |
| Abdomen yellow | 985 | **556** | 1349 | 752 |

Code for MCMCglmm R analysis

#Here we show the generic code used for all analyses using ‘trait’ in place of the relevant variable name.

#Priors for estimates of heritability

prior2=list(R=list(V=1, fix=1), G=list(G1=list(V=1, nu=1000, alpha.mu=0,alpha.V=1)))

prior2.2=list(R=list(V=1, fix=1), G=list(G1=list(V=1, nu=1000, alpha.mu=0,alpha.V=1),G2=list(V=1, nu=1000, alpha.mu=0,alpha.V=1)))

prior2.3=list(R=list(V=1, fix=1), G=list(G1=list(V=1, nu=1000, alpha.mu=0,alpha.V=1),G2=list(V=1, nu=1000, alpha.mu=0,alpha.V=1),G2=list(V=1, nu=1000, alpha.mu=0,alpha.V=1)))

#Four models per trait to estimate heritability

trait.MCMC=MCMCglmm(trait~1, random=~animal, ginverse=list(animal=Sinv), data=ped.data, family="ordinal", prior=prior2, nitt=205000, thin=100, burnin=50000, verbose=F)

trait.MCMC2=MCMCglmm(EB~1, random=~animal + Year, ginverse=list(animal=Sinv), data=ped.data, family="ordinal", prior=prior2.2, nitt=205000, thin=100, burnin=50000, verbose=F)

trait.MCMC2.1=MCMCglmm(trait~1, random=~animal + Dam, ginverse=list(animal=Sinv), data=ped.data, family="ordinal", prior=prior2.2, nitt=205000, thin=100, burnin=50000, verbose=F)

trait.MCMC3=MCMCglmm(trait~1, random=~animal + Year + Dam, ginverse=list(animal=Sinv), data=ped.data, family="ordinal", prior=prior2.3, nitt=205000, thin=100, burnin=50000, verbose=F)

#Bivariate animal model

trait1.trait2.var<matrix(c(var(ped.data$trait1,na.rm=TRUE),0,0,var(ped.data$trait2,na.rm=TRUE)),2,2)

prior.trait1.trait2<-list(G=list(G1=list(V=trait1.trait2.var/2,n=2)),R=list(V=trait1.trait2.var/2,n=2))

trait1.trait2.model<MCMCglmm(cbind(trait1,trait2)~trait1, random=~us(trait):animal, rcov=~us(trait):units, family=c("ordinal","ordinal"), ginverse=list(animal=Sinv), data=ped.data, prior=prior.trait1.trait2, nitt=205000, thin=100, burnin=50000,verbose=FALSE)
